# Idiopathic combined, autoantibody-mediated ADAMTS-13/factor H deficiency in thrombotic thrombocytopenic purpura-hemolytic uremic syndrome in a 17-year-old woman: a case report

**DOI:** 10.1186/1752-1947-5-598

**Published:** 2011-12-29

**Authors:** Daniel Patschan, Peter Korsten, Arne Behlau, Radovan Vasko, Malte Heeg, Nadera Sweiss, Gerhard A Müller, Michael Koziolek

**Affiliations:** 1 Department of Nephrology and Rheumatology, University Medicine Göttingen, Germany; 2 Section of Rheumatology, Department of Medicine, University of Chicago, Chicago, IL, USA

## Abstract

**Introduction:**

Thrombotic thrombocytopenic purpura-hemolytic uremic syndrome is a life-threatening condition with various etiopathogeneses. Without therapy approximately 90% of all patients die from the disease.

**Case presentation:**

We report the case of a 17-year-old Caucasian woman with widespread hematomas and headache. Due to hemolytic anemia, thrombocytopenia, and schistocytosis, thrombotic thrombocytopenic purpura-hemolytic uremic syndrome was suspected and plasma exchange therapy was initiated immediately. Since her thrombocyte level did not increase during the first week of therapy, plasma treatment had to be intensified to a twice-daily schedule. Further diagnostics showed markedly reduced activities of both ADAMTS-13 (a disintegrin and metalloproteinase with a thrombospondin type 1 motif, member 13 - also known as von Willebrand factor-cleaving protease) and factor H. Test results for antibodies against both proteins were positive. While plasma exchange therapy was continued, rituximab was given once weekly for four consecutive weeks. After the last dose, thrombocytes and activities of ADAMTS-13 and factor H increased into the normal range. Our patient improved and was discharged from the hospital.

**Conclusions:**

Since no clinical symptoms/laboratory findings indicated a malignant or specific autoimmune-mediated disorder, the diagnosis made was thrombotic thrombocytopenic purpura-hemolytic uremic syndrome due to idiopathic combined, autoantibody-mediated ADAMTS-13/factor H deficiency.

## Introduction

Thrombotic thrombocytopenic purpura-hemolytic uremic syndrome (TTP-HUS) can be diagnosed if a patient has combined hemolytic anemia, schistocytosis, and thrombocytopenia [1]. If untreated, approximately 90% of patients die from the syndrome. A number of different entities of TTP-HUS have been identified. In recent years, the pathogenesis has been elucidated in several subtypes of TTP-HUS. The 'classical' post-infectious TTP-HUS develops after intestinal infection with either Shigatoxin-producing *Escherichia coli *or *Shigella dysenteriae *[2,3]. However, defects of certain plasma proteins have been shown to be associated with TTP-HUS. A lack of ADAMTS-13 (a disintegrin and metalloproteinase with a thrombospondin type 1 motif, member 13 - also known as von Willebrand factor-cleaving protease), a protease responsible for degrading von Willebrand factor precursors, can cause severe cases of TTP-HUS [4]. Another group of defects is characterized by complement-mediated lysis of microvascular endothelial cells in kidney, brain, and other organs, respectively. The most frequent defect is factor H deficiency, which accounts for 15% of all TTP-HUS cases [5,6]. The treatment of choice of TTP-HUS is plasma exchange [1]. This recommendation results from the fact that mortality of untreated TTP-HUS is unacceptably high.

Here, we report a 17-year-old woman who presented with idiopathic combined autoantibody-mediated ADAMTS-13/factor H deficiency.

## Case presentation

A 17-year-old Caucasian woman presented to our facility with a history of fatigue, easy bruising and headache for two weeks. The headache was diffuse, dull and did not respond to oral ibuprofen at a dose of 400 mg up to three times daily. There were no associated visual symptoms, nausea or vomiting. There was no history of excessive menstrual bleeding, sexually transmitted disease or use of recreational or illicit drugs. The easy bruising was attributed initially to canoeing with some friends two days prior to admission. She had no history of trauma, no known allergies and denied recent medication use (other than ibuprofen). Our patient lived with her parents and a younger brother who did not have any known illnesses, and specifically no bleeding disorders. She was in her last year at high school and ate a normal diet. A physical examination showed a woman of Caucasian origin in a mildly obese nutritional state (64.8 kg) and appropriate appearance for her age. Heart, lung and abdominal examination results were unremarkable. Skin examination results revealed hematomas of different sizes ranging from 2.5 to 10.1 mm involving the arms, legs, and trunk. Laboratory test results from a local hospital revealed anemia with a hemoglobin level of 7.2 g/dL (normal range 11.5 to 15 g/dL) and thrombocytopenia of 19 × 10^6 ^cells/μL (normal range 150 to 350 × 10^6 ^cells/μL), for which our patient was referred to our center. Laboratory findings at the University Hospital of Göttingen showed severe anemia (hemoglobin at 6 g/dL), thrombocytopenia (17.7 × 10^6 ^cells/μL), an increase in lactate dehydrogenase (LDH) activity (963 U/L, reference < 350 U/L), and negative haptoglobin (< 0.2 g/L, normal range 0.45 to 2.05 g/L). Her schistocyte count was 10‰ (< 5‰). Her complement activity was reduced (C3 0.76 g/L, normal range 0.9 to 1.8 g/L; C4 0.06 g/L, normal range 0.1 to 0.4 g/L).

TTP-HUS was suspected given the anemia, thrombocytopenia, elevated LDH activity, non-measurable haptoglobin, and the presence of schistocytes in the peripheral smear. Our patient was referred to the intensive care unit for immediate plasma exchange therapy. For initial plasma exchange therapy, our patient received 250 mg prednisolone intravenously daily for three days, combined with 0.5 L of fresh frozen plasma. Plasma exchange therapy was started the next morning (approximately six hours after admission). The plasma volume to be exchanged per individual treatment session was calculated to be 40 mL/kg. Therapy efficacy was monitored by daily measurement of her thrombocyte levels. Although plasma exchange treatment was performed every day, her thrombocyte level decreased further, reaching 5 × 10^6 ^cells/μL at day 12 after admission. There was no evidence of bacterial, viral or parasitic infection. One week after admission our patient became confused, and developed disorientation and aggressiveness. Magnetic resonance scan results did not show any abnormalities. A computed tomography (CT) scan of her thorax, abdomen, and pelvis and blood tests for anti-nuclear antibodies (ANAs), anti-neutrophil cytoplasmic antibodies (ANCAs), and rheumatoid factor were performed [7]. The test results did not show any relevant abnormality. The only relevant finding was decreased complement activity. Around this time, the results from further serological analysis became available, showing a dramatically reduced activity of both ADAMTS-13 and factor H. Antibodies against the two proteins were positively detected. Therefore, and since increased activity of the disease was apparent, plasma exchange therapy was intensified to a twice-daily schedule. Her thrombocyte count remained stable at a very low range over the following eight days, with a minimum of 1 × 10^6 ^cells/μL at day 18 after the first plasma exchange. Our patient did not receive thrombocytes or coagulation factors. Intravenous rituximab, a chimeric monoclonal antibody against CD20, was initiated at a dose of 375 mg/kg/week, with four consecutive administrations [8]. At the same time, glucocorticoid therapy was continued with 1 mg/kg daily orally. Steroids were tapered over the following four months. Plasma exchange treatment was continued once to twice daily but was not performed on the days after rituximab infusion. Her thrombocyte count reached its first maximum at day 36 (314 × 10^6 ^cells/μL) and our patient's mental issues resolved completely. The last of the four infusions of rituximab was given at day 42 after admission; the last plasma exchange was performed three days later. Two days later, her thrombocyte count reached its second maximum of 344 × 10^6 ^cells/μL. Thereafter, thrombocytes decreased transiently (128 × 10^6 ^cells/μL), followed by an increase to 228 × 10^6 ^cells/μL on the day of discharge from hospital.

**Figure 1:**
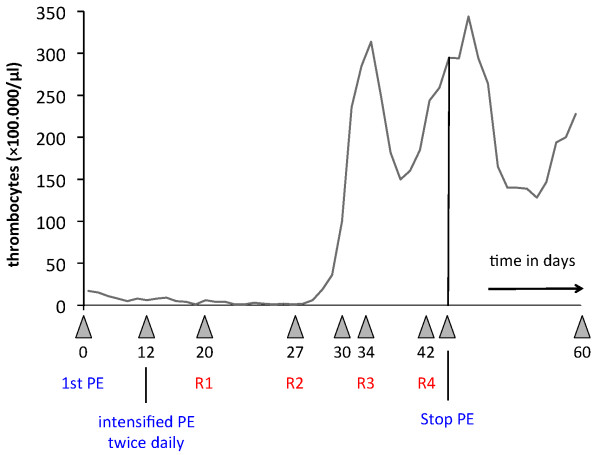
**Thrombocyte count over a period of 60 days**. After 12 days of plasma exchange treatments performed daily, therapy was intensified to a twice-daily schedule. Rituximab was administered for the first time at day 20 and then the drug was administered once weekly with a total of four dosages [8].

Figure 1 summarizes the thrombocyte count over her whole stay in the Department of Nephrology. Our patient's renal function was not affected at any given time and she did not exhibit any sediment abnormalities. Protein analysis, performed during the first two days after admission, showed low-grade proteinuria of mixed (that is, glomerular and tubular) origin. The activities of ADAMTS-13 and factor H were re-evaluated before our patient was discharged. Both enzymes were within normal ranges and antibody test results were negative. Table 1 shows the results from the initial blood analysis and from the day before discharge.

**Table 1:** Laboratory findings on the days of admission and a day before discharge

Parameter	Day of admission	Day before discharge
Hemoglobin (reference: 11.5 to 15.0 g/dL)	6	12
Thrombocytes (reference: 150 to 350 × 10^6 ^cells/μL)	17.7	228
Lactate dehydrogenase (reference: < 350 U/L)	963	237
Haptoglobin (reference: 0.45 to 2.05 g/L)	< 0.2	0.69
Fragmentocytes (reference: < 5‰)	10	< 5
Creatinine (reference: 0.55 to 1.02 mg/dL)	0.89	0.78

Since our patient's discharge she has continued to be asymptomatic with normal blood counts at 12-month follow-up. Our patient did not develop any infectious complications during the follow-up period.

In order to identify a possible genetic background for our patient's disease, her two parents and brother were screened for ADAMTS-13 and factor H activities, and for the concentrations of the respective antibodies to these factors. The results were completely negative (data not shown).

## Discussion

The coincidence of combined ADAMTS-13/factor H deficiency confirms the presence of TTP-HUS. Since no genetic background was identified to be associated with the enzyme deficit, the diagnosis to be made in our patient was idiopathic TTP-HUS due to combined (autoantibody-mediated) ADAMTS-13/factor H deficiency.

TTP-HUS can be diagnosed if a patient has combined hemolytic anemia, schistocytosis, and thrombocytopenia [1]. In fact, these findings legitimize immediate plasma exchange therapy even if no other obvious cause can be identified [1]. Such a recommendation results from the fact that mortality of untreated TTP-HUS is unacceptably high (up to 90%). Blood samples for further diagnostic steps should be drawn before the first plasma exchange therapy. As soon as plasma exchange therapy has been initiated, further diagnostic steps have to be made in order to recognize diseases that can mimic TTP-HUS. First and foremost, infectious diseases have to be excluded. Systemic bacterial infections, and in particular sepsis are frequently associated with the presence of schistocytes and thrombocytopenia [9]. This, in most cases, results from disseminated intra-vascular coagulation. A subsequent lack of pro-coagulatory proteins can cause bleeding complications with anemia, which can also result from microvascular erythrocyte fragmentation. In some situations it may become complicated to clearly differentiate between TTP-HUS and alternative diagnoses. This is the case (a) in systemic lupus erythematosus (SLE) with or without simultaneous anti-phospholipid syndrome (APS), in (b) systemic sclerosis (SS) with renal crisis, and (c) in malignant hypertension.

The question of whether a patient has TTP-HUS becomes even more complicated in pregnancy. Approximately one to three in every 300 pregnant women develops HELLP (hemolysis, elevated liver enzymes, low platelet count) syndrome [10]. Besides hemolysis and thrombocytopenia, schistocytosis is a hallmark of HELLP syndrome. TTP-HUS during pregnancy is almost indistinguishable from HELLP syndrome, and requires a completely different therapeutic approach (plasma exchange treatment).

**Figure 2:**
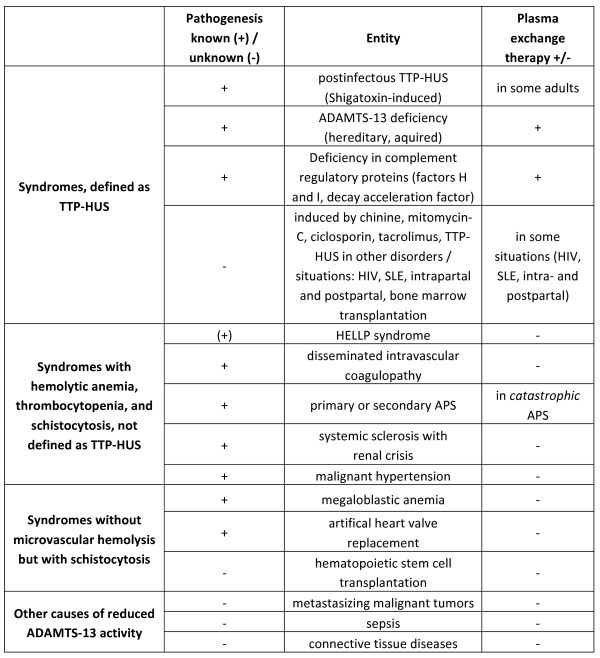
**Differential diagnosis of thrombotic thrombocytopenic purpura-hemolytic uremic syndrome (TTP-HUS)**.

Next, the different types of 'classical' TTP-HUS have to be taken into consideration. Post-infectious TTP-HUS, either induced by enterohemorrhagic *E. coli *(serotype O 157 or O 104) or by *S. dysenteriae*, is more frequently seen in children than in adults [11]. It does not require plasma exchange treatment. Further perpetuating factors in TTP-HUS are deficiencies of complement regulatory proteins (factor H, factor I, and decay-accelerating factor [5]). It has been considered that impaired inhibition of complement proteins may trigger complement-induced damage of host cells. In this process, endothelial damage may be responsible for microthrombogenesis in small arteries and capillaries, which subsequently induces thrombocytopenia, hemolysis, and schistocytosis. Finally, in the setting of reduced ADAMTS-13 activity, other causes of diminished enzyme activity not related to TTP-HUS have to be ruled out. In fact, low ADAMTS-13 is not specific for TTP-HUS [4]. Reduced concentrations of the protein, leading to increased circulating levels of ultra-large von Willebrand factor (ULVWF) have been shown in a subset of patients with sepsis. Impaired von Willebrand factor cleaving activity of ADAMTS-13 was also demonstrated in patients with metastasizing malignant tumors. In addition, systemic connective tissue diseases are other conditions that can be associated with low but detectable levels of ADAMTS-13. Figure 2 summarizes the differential diagnosis of TTP-HUS.

Although a number of different entities of TTP-HUS have been identified, which require different therapeutic approaches, it is mandatory to initiate plasma exchange treatment as soon as thrombocytopenia and hemolytic anemia with simultaneous schistocytosis are diagnosed [1]. The only exception is suspected TTP-HUS in children who have had diarrhea prior to diagnosis. In these cases, post-infectious TTP-HUS or childhood Shigatoxin-associated TTP-HUS must be assumed. Therapeutic measures include fluid administration and dialysis treatment in severe cases. In most children Shigatoxin-associated TTP-HUS recovers spontaneously. In adults, the situation with Shigatoxin-associated TTP-HUS is quite different. Although the pathogenesis is the same, renal and neurological involvement are usually more severe [12]. In addition, older patients have a mortality rate of approximately 90%, which is at least 15-fold higher than in children [13]. Even today, there are no prospective, randomized trial results available that resolve the question of whether plasma exchange therapy, either by simply infusing plasma or by plasma exchange, may be of benefit in Shigatoxin-associated TTP-HUS in adults [14]. Regardless, plasma exchange therapy can serve as an option, since comparative analyses of patients treated without plasma versus patients treated with plasma suggest a significantly decreased overall mortality rate in the latter [14]. Therefore, Ruggenenti and colleagues [14] recommended plasma infusion or exchange in adult patients with Shigatoxin-associated TTP-HUS if severe renal insufficiency is present and/or central nervous system is involved.

In TTP-HUS caused by either ADAMTS-13 or by complement deficiency, plasma exchange therapy is the treatment of choice. Initially, plasma exchange therapy should be performed daily and treatment should be monitored by measuring the thrombocyte count. In most cases, initial plasma administration should be accompanied by glucocorticoids. Whether and when the patient will respond to treatment is difficult to predict. In cases without any response to daily plasma exchange therapy, the regimen can be intensified to a twice-daily schedule [14]. Thus, recycling of infused plasma is minimized. Another alternative is to apply the cryosupernatant fraction of plasma. The cryosupernatant remains after removal of a cryoprecipitate, containing the largest amount of ULVWF, fibrinogen, fibronectin, and fibrin split products, respectively. This approach has successfully been used in a few patients with refractory TTP-HUS but its use is limited by restricted availability.

If intensified plasma exchange therapy or the administration of cryosupernatant does not sufficiently control disease activity, further pharmaceutical interventions can be initiated. First, and as already pointed out, glucocorticoids are widely used for initial therapy. Perotti and colleagues found remission rates of approximately 75% in patients with TTP-HUS who received combined treatment with intravenous methylprednisolone (2 mg/kg/day) and daily plasma exchange. However, to date, no systematic clinical trial has compared this regimen with plasma exchange alone. Patients with milder manifestations of TTP-HUS, in particular patients with no neurological symptoms, may respond to glucocorticoids alone [15]. Another drug that has occasionally been used in refractory or relapsing TTP-HUS is vincristine. Cyclophosphamide has been used as both daily dosing and pulsed therapy [16]. The common characteristic of drugs such as vincristine and cyclophosphamide is their potency to suppress the activity of lymphocytes. This mechanism is most likely responsible for the effectiveness of the substances in the treatment of refractory TTP-HUS. It must be assumed that suppression of autoantibody-producing B cell clones and of their respective descendants, the plasma cells, inhibits further autoantibody production and thus further immune-mediated depletion of ADAMTS-13 and complement regulatory proteins, respectively. The same rationale legitimates the administration of rituximab. The first mention of rituximab as a therapeutic option in cases of refractory TTP-HUS was published in 2002 [17]. Two patients, both women, with TTP-HUS refractory to plasmapheresis, glucocorticoids, and vincristine responded to the treatment after adding rituximab. Since then, the drug has been successfully used in resistant TTP-HUS with relevant response rates [18]. The mechanism of action of rituximab appears to be suppression of antibody-producing B cells [19], but effects on regulatory T cells have also been described which might attenuate the inflammatory response in those cases that are not merely triggered by antibodies [20]. An even newer therapeutic tool is the substance eculizumab. The drug acts as inhibitor of complement factor C5, which is activated by cleaving through the factor complex C3bBbC3b. Eculizumab has initially been used to treat patients with paroxysmal nocturnal hemoglobinuria (PNH) [21]. In 2008, Nürnberger and colleagues reported the first successful use of the drug in atypical (complement-mediated) hemolytic-uremic syndrome [22]. Since then, the substance has been administered with convincing response rates in patients who had an atypical type of TTP-HUS [23-26]. It should be noted that the mode of action of eculizumab in TTP-HUS is most likely not the inhibition of complement-induced red blood cell lysis but the inhibition of complement-mediated endothelial cell damage with subsequent microthombus formation.

Our patient showed combined deficiency of ADAMTS-13 and complement factor H. With regard to the complement defect, eculizumab was initially discussed as a therapeutic alternative. But, with the presence of autoantibodies against both proteins, a B-cell depleting therapy seemed much more promising. In fact, our patient responded with complete and, to date, stable remission. Regarding the heterogenous nature of the defect, any prediction of potential relapses is impossible.

## Conclusions

Our patient had idiopathic combined, autoimmune-mediated ADAMTS-13/factor H deficiency. Although, to the best of our knowledge, such a defect has never been reported before in the literature, it is important to recognize that therapy resistance in TTP-HUS can result from combined deficiencies in plasmatic regulators. Regardless, any secondary cause of TTP-HUS must be ruled out before drugs such as rituximab and eculizumab are administered to these patients. The use of rituximab in patients without any evidence of autoantibody production cannot be recommended. Regular follow-up with patients is needed to detect infections shortly after treatment, and in the long-term to detect secondary malignancies or relapses.

## Consent

Written informed consent was obtained from the patient's legal guardian for publication of this case report. A copy of the written consent is available for review by the Editor-in-Chief of this journal.

## Competing interests

The authors declare that they have no competing interests.

## Authors' contributions

DP is currently our patient's physician. DP and KP wrote the article. AB, RV, and MH were responsible for treating our patient in the intensive care unit. NJS helped in the preparation of the manuscript. GAM participated in writing the article. MK also participated in writing the article and helped to collect all necessary data. All authors read and approved the final manuscript.
